# Protocol for a randomised controlled trial of a school based cognitive behaviour therapy (CBT) intervention to prevent depression in high risk adolescents (PROMISE)

**DOI:** 10.1186/1745-6215-11-114

**Published:** 2010-11-29

**Authors:** Paul Stallard, Alan A Montgomery, Ricardo Araya, Rob Anderson, Glynn Lewis, Kapil Sayal, Rhiannon Buck, Abigail Millings, John A Taylor

**Affiliations:** 1 Department for Health, University of Bath, UK; 2 School of Social and Community Medicine, University of Bristol, UK; 3 Academic Unit of Psychiatry, Bristol University, UK; 4 Peninsula Medical School, University of Exeter, UK; 5 School of Community Health Sciences, Nottingham University, UK

## Abstract

**Background:**

Depression in adolescents is a significant problem that impairs everyday functioning and increases the risk of severe mental health disorders in adulthood. Relatively few adolescents with depression are identified and referred for treatment indicating the need to investigate alternative preventive approaches.

**Study Design:**

A pragmatic cluster randomised controlled trial evaluating the effectiveness of a school based prevention programme on symptoms of depression in "high risk" adolescents (aged 12-16). The unit of allocation is year groups (n = 28) which are assigned to one of three conditions: an active intervention based upon cognitive behaviour therapy, attention control or treatment as usual. Assessments will be undertaken at screening, baseline, 6 months and 12 months. The primary outcome measure is change on the Short Mood and Feeling Questionnaire at 12 months. Secondary outcome measures will assess changes in negative thoughts, self esteem, anxiety, school connectedness, peer attachment, alcohol and substance misuse, bullying and self harm.

**Discussion:**

As of August 2010, all 28 year groups (n = 5023) had been recruited and the assigned interventions delivered. Final 12 month assessments are scheduled to be completed by March 2011.

**Trial Registration:**

ISRCTN19083628

## Background

Epidemiological studies suggest that over a six month period up to 8% of adolescents suffer from a major depressive disorder [1]. Cumulative rates indicate that up to 20% will experience at least one clinically depressive episode by the age of 18 [2]. Adolescent depression causes significant impairment, impacts on developmental trajectories, interferes with educational attainment, and increases the risk of attempted and completed suicide as well as major depressive disorder in adulthood [1,3-5]. In addition, sub-threshold depressive symptoms in adolescence carry a similar risk to major depression for developing depression and suicidal behaviours later in life [6]. Whilst approximately 50% of children are estimated to spontaneously recover, for the remaining half symptoms persist and significantly impair functioning [7]. It is therefore particularly concerning to note that depression in adolescents often remains unrecognised and untreated [8,9].

Reducing rates of adolescent depression is an important public health issue and has resulted in growing interest in the development of preventative interventions. A recent review identified almost 30 different depression prevention programmes which can be delivered in schools [10]. These are either provided universally to all young people irrespective of risk status or in more targeted ways, i.e. to adolescents at increased risk of developing depression or already demonstrating mild/moderate problems [11]. The inclusivity of universal programmes minimises negative effects of stigma and labelling, resulting in higher participation rates and lower rates of dropout [12-14]. In addition, universal programmes provide opportunities for primary prevention, i.e. reducing the onset of new problems, through the promotion of skills which develop good psychological health and resilience. However, from an economic perspective, interventions are provided for children who may not ever require them. Furthermore, in terms of effectiveness, targeted interventions typically result in greater reductions in depressive symptoms [2,10,15]. This may be due to initial levels of depressive symptoms in targeted groups being higher resulting in greater post intervention reductions [15]. Whilst universal approaches might have a more limited effect on individuals, they offer the potential to reduce far more disorders in the population as a whole than a highly effective targeted approach [16,17].

Despite the proliferation of school based depression prevention programmes, current research suffers from significant methodological limitations [2,10,15,18]. Studies are often underpowered, medium term follow-ups are lacking, and few have included comparisons with other active interventions. Most are efficacy studies assessing interventions under highly controlled conditions and no trials have been undertaken within the UK educational system. In terms of programme content, those based upon cognitive behaviour therapy (CBT) tend to show most promise. The majority of CBT based programmes produce immediate reductions in depression scores although these gains are not always maintained at follow-up. There are also considerable differences between programmes in the number of sessions, core components and delivery (e.g. delivered by teachers or health professionals). Variations in effectiveness within similar programmes suggest the importance of mediating and moderating factors.

One promising universal depression prevention programme is the Australian Resourceful Adolescent Programme (RAP). In the initial efficacy study, 260 adolescents were assigned to RAP, RAP plus family involvement or a no intervention group [19]. In terms of reach, 85% of the eligible cohort took part and attrition was low at 5.8%. Adolescents who received either version of RAP reported significantly lower levels of depressive symptomatology at post intervention and 10 month follow-up compared with the no intervention group.

In a New Zealand adaptation of the programme, RAP-Kiwi, 392 students were assigned to either RAP-Kiwi or an attention placebo condition [20]. Recruitment rates were again high (73%), attrition low (9% attrition at 6 months) and depression scores were significantly lower in the RAP group post intervention. The results were less clear at the 18 month follow-up where differences on one of the two depression scales were maintained.

Finally, a large multi-site randomised controlled effectiveness trial in Australia of RAP involving 2664 students from 12 schools has recently been undertaken [21]. RAP participants recorded significantly lower levels of depressive symptoms than those in the control condition at both post-intervention and 12-month follow-up. Based on initial depression scores, significantly more (49%) of the at-risk students in the RAP condition moved into the healthy category at post-intervention compared with the control group (35%). This difference was maintained at 12 month follow-up. In a subsequent qualitative evaluation with 109 young people, 61% of girls and 46% of boys were able to identify specific examples where they had used skills learned during RAP [21]. The authors concluded that within the context of a real world effectiveness study RAP appears to positively affect the health status of "at risk" students.

In conclusion, there is evidence to suggest that school based preventive approaches can have a short term effect upon adolescent depression. However, most trials are small underpowered efficacy studies which have rarely involved comparisons with other groups.

## Methods and design

### Aim of the study

The primary aim of this study is to examine whether a school based CBT programme (Resourceful Adolescent Programme) is more effective than an attention control or usual school curriculum in reducing depressive symptoms in "high risk" adolescents attending UK schools.

### Design

The study is a pragmatic cluster randomised controlled trial comparing the effectiveness of three school based interventions: CBT, attention control or usual school curriculum. The arms are summarised in Table 1.

**Table 1:** Arms of the PROMISE Randomised Controlled Trial

Study Arm	Content	Delivery
Treatment as usual	Normal school curriculum	One school staff

Attention control	Normal school curriculum	One school staff (leading sessions) plus two facilitators

Cognitive Behaviour Therapy	Resourceful Adolescent Programme	Two facilitators (leading sessions) plus one school staff

Interventions are delivered in schools to whole classes of children as part of the school curriculum. Assessments are administered at initial screening, baseline (2 weeks after screening), 6 months and 12 months.

### Participants and Eligibility

Interventions are provided as part of the school personal, social and health education (PSHE) curriculum. All pupils in years 8-11 (12-16 years old) are eligible to participate. Children are ineligible if they are not attending school (e.g. long term sickness, excluded from school, educated elsewhere) or do not participate in PSHE for religious or other reasons.

### Ethical Approval and Consent

The study was approved by the School for Health Research Ethics Approval Panel at the University of Bath. Consent/assent involves three stages. Firstly, eligible schools were provided with information about the study and interested head teachers were required to provide written confirmation that their school wanted to participate. Secondly, information was posted to the parents of all eligible children at their home address. Parents were invited to return a form opting out of the study if they did not wish their child to complete the study assessments. Finally, children were provided with information about the study and were required to provide written assent before completing assessments if they decided to opt in. Dual carer/child consent/assent was required for assessment completion.

The ongoing conduct and progress of the trial is monitored by two independently chaired committees. The Data Monitoring and Ethics Committee (DMEC) and a Trial Steering Committee (TSC) meet at least annually.

### Recruitment

A list of 66 mixed non-denominational comprehensive secondary schools in five sites incorporating urban and rural areas (Nottingham, Nottinghamshire, Wiltshire, Bath and North East Somerset and Bristol) was complied from local authority information. Project information sheets were sent to the Head Teachers and PSHE leads at each school. Meetings were arranged with staff at those schools who expressed interested and participation required a signed letter from the head teacher.

Nine schools were recruited, one for the pilot study and 8 for the main trial. In the main trial, one is a new school and does not have a Year 11 group. A further two schools could not include their Year 11 groups and one of these was also unable to include their Year 10 group. In total, the 8 schools recruited for the main trial comprise 28 year groups, 222 classes, and involves 5,768 young people.

### Randomisation

Allocation of year groups was undertaken once all schools were recruited. Trial arms were balanced with respect to key characteristics by calculating an imbalance statistic for a large random sample of possible allocation sequences [22]. The variables used for balancing were school, available year groups, number of students, number of classes, PSHE frequency and timetabling of PSHE lessons. A statistician with no other involvement in the study randomly selected one sequence from a subset with the most desirable balance properties.

### Participant Classification

All young people in a year group receive the intervention to which the year group is assigned (i.e. universal delivery). However, the primary aim of the study is to determine the effects on adolescents identified as at "high risk" of depression. Classification of risk is based upon two criteria, i.e. raised levels of depressive symptoms and continuity over time. Research with clinical and community samples demonstrates that adolescents who fulfil diagnostic criteria for depression achieve mean scores on short forms of the MFQ in the range of 7.01 - 11.95 compared with 3.24 - 4.68 for those who are not depressed [23-26]. A score of 5 was chosen to categorise adolescents as either low (< 5) or high risk (≥ 5). To account for transient changes in mood, MFQ scores are collected on two separate occasions approximately two weeks apart. Adolescents, who display persistent symptoms, i.e. scoring ≥ 5 on both occasions, will form the high risk group and we predict that approximately 20% will fall within this category.

### Interventions

#### Resourceful Adolescent Programme (CBT)

The Resourceful Adolescent Programme (RAP) consists of 9 modules and 2 booster sessions, each lasting approximately 50-60 minutes. The modules can be flexibly delivered in order to fit within the school timetable. Each programme is led by two trained facilitators working alongside the class teacher.

RAP is based upon cognitive behavour therapy (CBT) but also draws upon ideas from interpersonal therapy and specifically targets low mood. Through the programme, young people are helped to recognise the importance of negative thoughts and low self-worth/image in the onset and maintenance of depression. Core components include psycho-education, identifying and challenging negative/dysfunctional thoughts, identifying personal strengths (thereby enhancing self-esteem/image), managing social problems, and learning to problem solve.

In the first session, young people are introduced to RAP and are helped to identify their existing strengths and personal resources, thereby emphasising the importance of developing and maintaining good self-esteem. Modules two and three are concerned with emotional recognition and management. Physiological symptoms associated with different emotional states are identified and young people are encouraged to practice and use a variety of strategies to manage any unpleasant feelings. The fourth and fifth modules focus upon cognitions and highlight helpful and unhelpful ways of thinking. Common negative ways of thinking (i.e. thinking traps) are identified and young people are provided with a framework for checking and reappraising their cognitions. Module six is concerned with problem solving and the development of a stepped approach to generate and evaluate possible solutions to challenges. The seventh module highlights the importance of support networks and who young people can approach to help them cope with problems. Modules eight and nine are concerned with conflict resolution and how helping to understand another person's perspective can lead to "win-win" solutions. Two additional booster sessions will be provided approximately 6 months later. These will provide opportunities to review all RAP skills and to practice applying them to a number of common difficulties.

### Attention Control

The attention control intervention involves similar time and contact as the CBT intervention. The class teacher leads and delivers their usual PSHE curriculum, but is joined by two facilitators who assist with delivering the lessons and engaging with young people. This controls for the non-specific effects of interventions that are considered important in studies of depression [27]. In the same way as RAP, the delivery of the Attention Placebo intervention is flexible to fit with the existing school PSHE programmes.

### Usual PSHE

Young people participate in the usual personal health and social education (PSHE) sessions provided by the school (i.e. treatment as usual). The sessions are provided solely by the teacher and do not involve any external input from the research team. The content of the usual PSHE sessions will be examined to determine any potential overlap with the active intervention.

### Facilitators

Two facilitators are provided for the RAP and attention control group. Facilitators have at least an undergraduate university degree (some also had postgraduate qualifications) in a relevant discipline. All have appropriate professional backgrounds, including experience of working with young people. Separate initial training and on-going supervision was provided for the facilitators in each group. Wherever possible, the two facilitators are scheduled to remain with their assigned classes for the duration of the intervention.

### Outcome Measures

#### Primary Outcome

The primary outcome measure is changes in the level of symptoms of low mood as determined by the Short Mood and Feelings Questionnaire (SMFQ) at 12 months after the start of the trial [23]. The SMFQ is a unifactorial scale with a robust single factor structure [28,29]. Criterion validity (i.e. ability to predict clinical diagnosis) has been established within both clinical [23] and community samples [25,30] and with children ranging in age from 7-16. The scale correlates well with other measures of depression, has good test/re-test reliability with higher scores tending to be associated with children who fulfil diagnostic criteria for clinical depression [23-25].

#### Secondary Outcomes

A range of secondary outcome measures are also being collected. Negative thoughts will be assessed by examining scores on the personal failure sub-scale of the Children's Automatic Thoughts Scale (CATS; [31]. The Rosenberg Self-Esteem Scale assesses changes in self-worth and self-acceptance [32]. The Revised Child Anxiety and Depression Scale (RCADS;[33] assesses changes in symptoms of DSM-defined anxiety disorders and major depression in children. Five sub-scales assess symptoms of generalised anxiety disorder, separation anxiety disorder, social phobia, panic disorder and major depressive disorder. The degree to which children feel accepted, valued respected and included in their school is assessed by the short form of the Psychological Sense of School Membership (PSSM) scale [34]. The relationship with friends is assessed by the Attachment Questionnaire for Children [35]. Finally, questions will assess the extent to which children have bullied others, been the victim of bullying; drunk alcohol; smoked cannabis; taken other drugs; experienced thoughts of self harm and whether they have harmed themselves.

### Economic Evaluation

Quality of Life is assessed by the EQ-5D. This is a standardised instrument to assess health outcomes and assesses 5 dimensions of health (mobility, self-care, usual activities, pain/discomfort anxiety/depression). Usage of health services is assessed via a modified (self completed) version of the Client Service Receipt Inventory [36] which measures the use of health, education and social care services over the previous 6 months.

Incremental cost-effectiveness analysis using the primary clinical outcome, (i.e. SMFQ) and cost-utility analysis (i.e. cost per QALY, on the basis of utility estimates derived from EQ-5D scores) for all included comparators over the 12-month time horizon of the trial will be undertaken. These analyses will be from a societal perspective, capturing and where possible valuing cost and other potential impacts of the intervention across the health, education and social care sectors. Sensitivity analysis will be used to express uncertainty in the cost-effectiveness estimates

### Power calculation

The pilot site (n = 711) provided estimates of ICC (0.025), mean year group size (n = 203) and consent rate (84%) and SMFQ standard deviation (4.9). Based on 80% consent, 80% retention and 20% of children being classified as "at risk", effect sizes in the range of 0.36-0.42 SDs are detectable with 80% power and 5% two-sided alpha with 20-27 year groups. These are equivalent to differences in SMFQ scores of around 2 points. A total of 28 year group groups have been randomised.

### Statistical analysis

Data analysis will be undertaken blinded to allocation. The quantitative data will be analysed and the study reported in accordance with the CONSORT guidelines for randomised controlled trials [37]. Using routine data, we will compare characteristics of participating schools with (a) schools that were invited and did not participate, (b) schools in England. Within the eight schools, we will also compare characteristics of participating students with those who did not take part in any PSHE sessions, and those who did not provide consent to complete study questionnaires. Using appropriate descriptive statistics, we will assess comparability of the three trial arms at baseline, and to describe the number and characteristics of participants who do not provide outcome date at follow up.

The primary outcome is the Short Mood and Feelings Questionnaire (SMFQ) 12 months after baseline. The primary analysis will be restricted to participants identified as 'high risk' based on their screening and baseline SMFQ scores (≥ 5 on both occasions).

The first stages will involve examination of the distribution of SMFQ 12-month scores. Scores for each of the trial arms will be described using mean (SD) or median (IQR) as appropriate and appropriate transformation of the data undertaken if required. The primary intention-to-treat (ITT) analysis will be an analysis of covariance that takes appropriate account of the clustered design and school structure, implemented using random effects linear regression models in MLwiN software. Depending on extent of missing primary outcome data, the primary analysis will be repeated using complete data set generated using multiple imputation. The two comparisons of primary interest are the RAP arm versus each of the two control arms, presented as between-group differences in means, 95% confidence intervals and p-values adjusted for multiple comparisons using Dunnett's correction. Covariates in the model will comprise SMFQ baseline score and the six variables used to balance the trial arms in the allocation procedure.

Although year group is the unit of allocation, PSHE sessions are delivered to classes nested within year group, and year groups are themselves nested within schools. We will therefore investigate the components of variance at individual, class, year group, and school level before determining the final model which will be at least a two-level model (individual + year group), and may be up to a four-level model.

Analysis of secondary outcomes will be undertaken using the same general approach as for the primary analysis, using linear or logistic regression models for continuous or binary outcomes as appropriate. As a pragmatic trial of intervention 'effectiveness' (rather than 'efficacy'), the primary ITT analysis makes no attempt to take account of actual intervention received. We will also investigate efficacy among participants who comply with the intervention using instrumental variable methods [38]. Finally, using appropriate interaction terms in regression models, we will undertake subgroup analyses of SMFQ continuous score at 12 months according to baseline (i) SMFQ (< 5, 5-10, ≥ 11), (ii) self-harm, (iii) alcohol/drug misuse, (iv)year group, (v) Family Affluence Scale

### Study Status

A pilot study involving one school started in December 2008 and was completed in January 2010. The main trial started in September 2009 and all schools have now been recruited. The 12 month follow-up assessments are scheduled to be completed by the end of March 2011.

## Competing interests

None of the authors have any competing interests arising from this research.

## Authors' contributions

PS, GL, AM, RA, RA & KS conceived the study and led the bid to secure funding for this work. They have contributed to the development of the protocol and are involved in managing and advising on the project. RB, AM & JT are the researchers employed on this project and have contributed to the development of the protocol and the drafting of this paper.
